# Trichostatin A, a histone deacetylase inhibitor, suppresses proliferation and epithelial–mesenchymal transition in retinal pigment epithelium cells

**DOI:** 10.1111/jcmm.12212

**Published:** 2014-01-23

**Authors:** Wei Xiao, Xiaoyun Chen, Xialin Liu, Lixia Luo, Shaobi Ye, Yizhi Liu

**Affiliations:** State Key Laboratory of Ophthalmology, Zhongshan Ophthalmic Center, Sun Yat-sen UniversityGuangzhou, China

**Keywords:** histone deacetylase inhibitor, proliferation, epithelial–mesenchymal transition (EMT), retinal pigment epithelium (RPE) cells, proliferative vitreoretinopathy (PVR)

## Abstract

The proliferation and epithelial–mesenchymal transition (EMT) of retinal pigment epithelium (RPE) cells are the major pathological changes in development of proliferative vitreoretinopathy (PVR), which leads to severe visual impairment. Histone deacetylases (HDACs)-mediated epigenetic mechanisms play important roles in controlling various physiological and pathological events. However, whether HDACs are involved in the regulation of proliferation and EMT in PRE cells remains unidentified. In this study, we evaluated the expression profile of HDAC family (18 genes) and found that some of class I and class II HDACs were up-regulated in transforming growth factor-β2 (TGF-β2)/TGF-β1-stimulated RPE cells. Tricostatin A (TSA), a class I and II HDAC inhibitor, suppressed the proliferation of RPE cells by G1 phase cell cycle arrest through inhibition of cyclin/CDK/p-Rb and induction of p21 and p27. In the meantime, TSA strongly prevented TGF-β2–induced morphological changes and the up-regulation of α-SMA, collagen type I, collagen type IV, fibronectin, Snail and Slug. We also demonstrated that TSA affected not only the canonical Smad signalling pathway but also the non-canonical TGF-β/Akt, MAPK and ERK1/2 pathways. Finally, we found that the underlying mechanism of TSA affects EMT in RPE cells also through down-regulating the Jagged/Notch signalling pathway. Therefore, this study may provide a new insight into the pathogenesis of PVR, and suggests that epigenetic treatment with HDAC inhibitors may have therapeutic value in the prevention and treatment of PVR.

## Introduction

Proliferative vitreoretinopathy (PVR) is a severe complication of retinal detachment (RD) and the most common cause of surgical failure in the RD treatment. It is characterized by formation of fibrotic membranes, which reduce the flexibility of retina, and further result in retinal redetachment and difficulty in retinal reattachment 1. Although advances in surgical techniques have reduced the PVR rate, it is still a significant issue in RD management.

The growing body of evidence shows that proliferation and EMT are two major pathological changes in development of fibrotic lesions on the retina 2,3. It is initiated by the rupture of the blood–retinal barrier, through which serum cytokines, growth factors and inflammatory cells penetrate into the vitreous cavity and/or sub-retinal space 3. Several kinds of cells, including hyalocytes, retinal müller glial cells, fibroblasts and macrophages, are involved in development of retinal fibrosis 4. Of note, RPE cells are the most important contributor in this process 5. Various cytokines are able to promote these cells to proliferate, migrate towards the vitreous body or intraretinal layers, produce extracellular matrix components and transform into fibroblast-like cells, which further results in the formation of pre- and sub-fibrous membranes 3. The fibrotic membranes can contract and cause retinal detachment and severe visual impairment. Transforming growth factor-β (TGF-β) has been proven to be the key cytokine in various fibrous diseases, such as liver cirrhosis, pulmonary fibrosis, systemic sclerosis and ocular fibrotic diseases 6. Transforming growth factor-β2, which is the major TGF-β isoform in the posterior segment of the eye, is overexpressed in the vitreous and proliferative membranes from patients with PVR 7,8. Therefore, agents capable of preventing the proliferation and EMT of RPE cells may be of great therapeutic value in preventing retinal fibrosis after reattachment surgery.

Histone deacetylases (HDACs)-mediated epigenetic mechanisms play important roles in control of various physiological and pathological events. Accumulating evidence has shown that HDACs are crucial targets in various diseases, including cancer, autoimmune and inflammatory diseases, and metabolic disorders 9. In the meantime, HDAC inhibitors are promising anticancer agents whose effects are correlated with the transcriptional regulation of specific cancer-related genes 10,11. To date, 18 human HDACs have been identified and grouped into four classes on the basis of their sequence homology and domain organization: class I HDACs (HDAC1, 2, 3 and 8), class II HDACs (HDAC4, 5, 6, 7, 9 and 10), class III HDACs (SIRT1, 2, 3, 4, 5, 6 and 7) and class IV HDAC (HDAC11) 12. Class II HDACs are further subdivided into class IIa (HDAC4, 5, 7, 9) and class IIb (HDAC6 and 10) forms 12. Recently, HDAC inhibitors are also shown to have an antifibrogenic effect in some organs, such as the liver, skin and lung 13. For instance, tricostatin A (TSA), a class I and II HDAC inhibitor, inhibited trans-differentiation of hepatic stellate cells into myofibroblasts 14, and also abrogated TGF-β2–induced EMT in skin fibroblasts, mouse hepatocytes and human renal epithelial cells 13,15,16. Phenylbutyrate, a weaker HDAC inhibitor, decreased collagen type I production in human pulmonary fibrosis 17. Despite their definite effect on EMT inhibition, the underlying mechanisms need to be clarified 13. Moreover, the function of HDAC inhibitor in RPE cells undergoing EMT is still unknown.

In this study, we identified, for the first time, that some of class I and class II HDACs were up-regulated in TGF-β2/TGF-β1–induced EMT in human RPE cells. Inhibition of HDAC activity with TSA strongly inhibited the proliferation of RPE cells by cell cycle arrest. Moreover, TSA significantly prevented TGF-β2–induced EMT through regulating not only the canonical Smad signalling pathway but also the non-canonical TGF-β/Akt, MAPK and ERK1/2 pathways. We also demonstrated that the underlying mechanism of TSA affects EMT through down-regulating the Jagged/Notch signalling pathway. Therefore, these results suggest that inhibition of HDAC activity may be beneficial for PVR treatment.

## Materials and methods

### Reagents and antibodies

Recombinant human TGF-β1 and TGF-β2 were purchased from Cell Signaling (Danvers, MA, USA). Tricostatin A (a class I and II HDAC inhibitor) was purchased from Sigma-Aldrich (Louis, MO, USA). Antibodies against HDAC1, HDAC2, HDAC5, cyclin D1, cyclin-dependent kinase (CDK) 4, CDK6, P21, P27, p-Rb, Jagged1, Notch3, p-Smad2/3, Snail, Slug, p-Akt, Akt, p38MAPK, p-p38MAPK, ERK1/2, p-ERK1/2, horse antimouse and goat anti-rabbit horseradish peroxidase (HRP)-conjugated secondary antibodies, Alexa Fluor 488–conjugated goat anti-rabbit and Alexa Fluor 555–conjugated donkey antimouse secondary antibodies were purchased from Cell Signaling Technology (Beverly, MA, USA). Antibodies against β-actin, α-SMA, collagen type I (Col I), collagen type IV (Col IV), vimentin and fibronectin (FN) were purchased from Abcam (Cambridge, UK). Cell counting kit-8 (CCK-8) was purchased from Dojindo (Shanghai, China).

### Cells culture and treatment

The human retinal pigment epithelial cell line ARPE-19 was kindly provided by Professor Fu Shang at the Laboratory for Nutrition and Vision Research (Boston, MA, USA), and cultured in DMEM containing 10% foetal bovine serum. The cells were grown to confluence at 37°C in a humidified atmosphere containing 5% CO_2_ and dissociated with 0.25% trypsin–0.02% ethylenediaminetetraacetic acid solution.

For TGF-β1, TGF-β2 and TSA treatments, the cells were cultured in six-well plates and treated with 5 ng/ml recombinant human TGF-β1 or TGF-β2 and various concentrations of TSA for different time-points.

### Cell proliferation assay

The proliferation of RPE cells was examined using CCK-8 kit according to the manufacturer's instructions. Briefly, RPE cells were seeded into 96-well plates at the density of 5 × 10^3^ cells/well with 100 μl of complete culture medium and cultured for 24 hrs. Cells were treated with various concentrations of TSA for 24, 48 and 72 hrs respectively. At the end of the treatment period, 10 μl of CCK-8 solution was added to each well and incubated for 2 hrs. The absorbance (A) at 450 nm was measured using a microplate reader.

### Cell apoptosis assay

Cells were seeded on six-well plates and treated with different doses of TSA for 72 hrs, and then cells were harvested, washed with PBS and stained with Annexin V-FITC/PI (Becton Dickinson, Franklin Lakes, NJ, USA) at room temperature (RT) for 15 min. Samples were analysed by flow cytometry.

### Cell cycle analysis

After 24 hrs incubation with TSA or dimethylsulphoxide (DMSO), cells were harvested and fixed in ice-cold 70% ethanol overnight at 4°C. The cells were centrifuged down and resuspended in 0.2 ml propidium iodide staining solution. After 45 min. of incubation at RT, the DNA content of cells was measured using a flow cytometry (BD Biosciences, San Jose, CA, USA).

### Real-time PCR analysis for gene expression

Total RNA was extracted from cells with Trizol reagent (Invitrogen, Carlsbad, CA, USA) according to the manufacture's instruction. cDNA was synthesized with a reverse transcription kit (Takara, Siga, Japan), using conditions recommended by the manufacturer. For quantitative analysis of mRNA expression, SYBR®PrimeScript™ RT-PCR kit (Takara) was used to amplify the target genes by the ABI Prism 7000 sequence detection system (Applied Biosystems, Foster City, CA, USA). Glyceraldehyde 3-phosphate dehydrogenase was used as an internal control.

### Immunofluorescence

Retinal pigment epithelium cells (1 × 10^5^) were grown on cover slips in six-well plates and treated as described previously. After fixed with acetone for 10 min. and permeabilized with 0.1% Triton X-100 for 5 min., cells were blocked with 1% bovine serum albumin for 1 hr. Then, they were incubated with different primary antibodies at 4°C overnight. On the next day, cells were incubated with Alexa Fluor 488–conjugated goat anti-rabbit or Alexa Fluor 555–conjugated donkey antimouse secondary antibodies (1:200) for 1 hr at RT. After washing with PBS containing 0.1% Tween-20 (PBST), cells were incubated with DAPI to stain nuclei. The slides were mounted with anti-fade fluorescent mounting medium and images were acquired by a fluorescence confocal microscope (LSM510; Carl Zeiss, Overkochen, Germany).

### Western blot analysis for protein expression

For total protein extraction, cells were lysed with 100 μl of RIPA lysis buffer with protease inhibitor cocktail. The cell lysates were collected after centrifugation and mixed with 5× SDS sample buffer. The samples were loaded and separated on 10% SDS-PAGE, and then transferred to PVDF membranes. The membranes were blocked in 5% non-fat milk and incubated with various primary antibodies at 4°C overnight. After washing with PBST, the membranes were incubated with HRP-conjugated secondary antibodies for 1 hr at RT. The bands on the membranes were visualized using chemiluminescence detection reagents. Densitometic analysis was conducted by Image J software 1.41 (National Institutes of Health, Bethesda, MD, USA). β-actin was used as loading control.

### Statistical analysis

Experiments presented in the figures were representative of three or more different repetitions. All data were expressed as mean ± SEM and analysed with SPSS 15.0 software (SPSS Inc., Chicago, IL, USA). One-way anova was used to compare differences among groups. A value of *P* < 0.05 was considered statistically significant.

## Results

### Expression of HDACs in TGF-β–induced EMT of RPE cells

**Figure 1:**
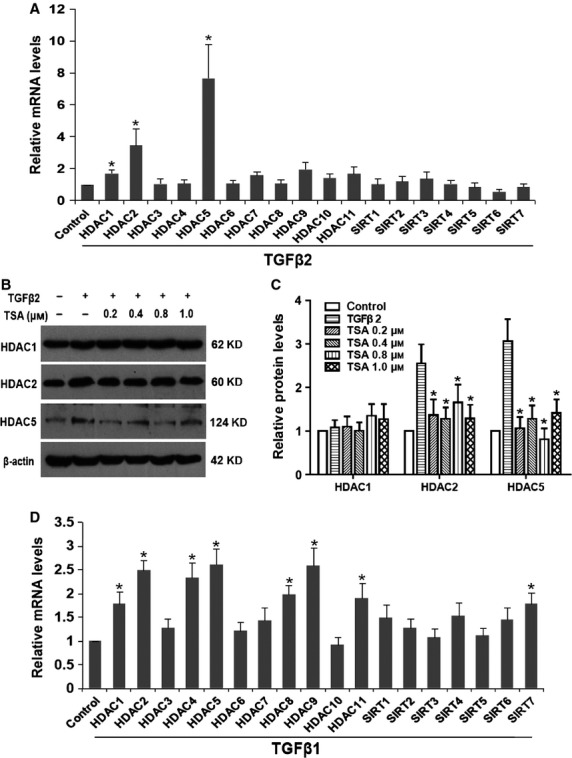
Expression of histone deacetylases (HDACs) in transforming growth factor-β (TGF-β)–induced epithelial–mesenchymal transition in retinal pigment epithelium (RPE) cells. (A) RPE cells were cultured in the presence or absence of TGF-β2 (5 ng/ml) for 24 hrs; the mRNA expression levels of HDAC family (18 isoforms) were detected by real-time quantitative PCR. Gene expression levels were normalized to the glyceraldehyde 3-phosphate dehydrogenase control. *P < 0.05 *versus* control group. (B) The protein expression levels of HDAC1, HDAC2 and HDAC5 in cells cultured in the absence or presence of TGF-β2 with tricostatin A (0.2, 0.4, 0.8 and 1.0 μM) or DMSO for 24 hrs were detected by western blot. (C) Quantification of protein levels from three independent experiments. *P < 0.05 *versus* TGF-β2 treated with DMSO group. (D) RPE cells were cultured with TGF-β1 (5 ng/ml) for 24 hrs; the mRNA expression levels of HDAC family were detected by real-time quantitative PCR. *P < 0.05 *versus* control group.

We first determined the expression pattern of HDAC family (18 genes) in TGF-β2–induced EMT in human RPE cells by real-time PCR. The result showed that TGF-β2 induced 6.70-, 2.47-, 0.97- and 0.70-fold increases in the abundance of HDAC5, HDAC2, HDAC9 and HDAC1 mRNA transcripts, while other HDAC family members were not significantly changed after 24 hrs of TGF-β2 treatment (Fig. 1A: *P < 0.05 *versus* control group). Similar alternations of HDAC5 and HDAC2 protein expression were observed by western blot, but no difference in HDAC1 was detected (Fig. 1B). Furthermore, the up-regulation of HDAC2 and HDAC5 induced by TGF-β2 were obviously inhibited by TSA (a class I and II HDAC inhibitor; Fig. 1B and C; *P < 0.05 *versus* TGF-β2 treated with DMSO group). In addition, we also assessed the expression of HDAC family in TGF-β1–induced RPE cells and found that HDAC1, HDAC2, HDAC4, HDAC5, HDAC8, HDAC9, HDAC11 and SIRT7 were up-regulated (Fig. 1D; *P < 0.05 *versus* control group). These results suggest that several family members of class I and class II HDACs are up-regulated in TGF-β2/TGF-β1–induced EMT in RPE cells.

### The HDAC inhibitor TSA inhibited the proliferation of RPE cells by cell cycle arrest

**Figure 2:**
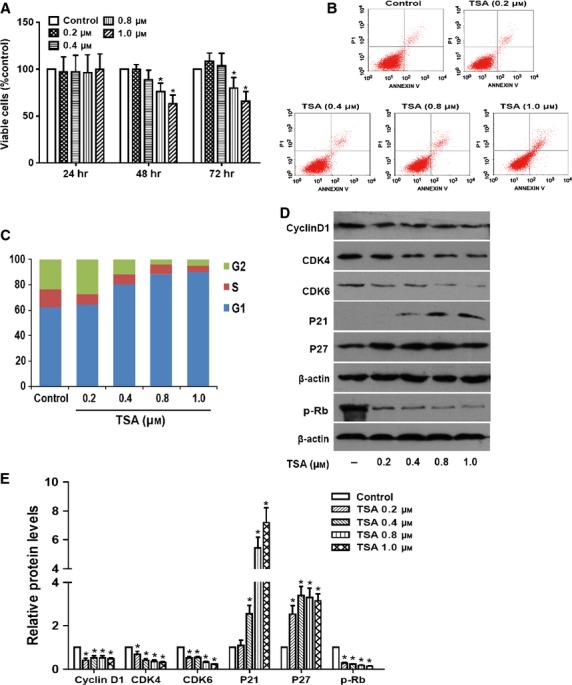
The histone deacetylases inhibitor tricostatin A (TSA) suppresses the proliferation of retinal pigment epithelium (RPE) cells by cell cycle arrest. (A) RPE cells were treated with TSA at various concentrations (0.2, 0.4, 0.8 and 1.0 μM) for 24, 48 and 72 hrs, and then the percentage of viable cells was determined using CCK-8 kit. (B) The apoptosis of cells was quantified by Annexin V-FITC/PI staining and determined using flow cytometry after treatment for 72 hrs. (C) Cell cycle analysis was quantified by PI staining followed by flow cytometry analyses after treatment for 24 hrs. Bar graphs represent the mean ± SEM of three independent experiments. (D) The protein expression levels of cyclinD1, CDK4 and CDK6, p-Rb, P21 and P27 were detected by western blot. (E) Quantification of protein levels from three independent experiments. *P < 0.05 *versus* control group.

Proliferation of RPE cells is the fundamental step during PVR, so we first examined the effect of the HDAC inhibitor TSA on growth of RPE cells. As shown in Figure 2A, TSA (0.8 and 1.0 μM) strongly exerted inhibitory effects on cell growth after treatment for 48 and 72 hrs (*P* < 0.05), but had no effect after 24 hrs (*P *>* *0.05). To determine whether the cell growth inhibition we observed was because of apoptosis induced by TSA, the apoptosis of cells was analysed using flow cytometry. The results showed that TSA higher than 0.8 μM of concentration led to cell death after treatment for 72 hrs (Fig. 2B). These results indicate that the inhibitory effect of low doses of TSA (0.4 and 0.8 μM) on growth inhibition is mainly because of suppressing proliferation, while higher dose of TSA (1.0 μM) can cause cell apoptosis. Furthermore, to determine if the anti-proliferative effect of TSA results from cell cycle arrest, cell cycle progression was next examined. Retinal pigment epithelium cells cultured with various concentrations of TSA for 24 hrs showed an accumulation of cells in the G1 phase of the cell cycle (from 62.1% to 90.1%), with concomitant decreases in the proportion of those in G2 (from 23.5% to 4.7%) and S phases (from 14.4% to 5.2%; Fig. 2C). To investigate the mechanism under cell cycle arrest, the effect of TSA on the expression of cell cycle–regulated proteins was examined by western blot. As expected, TSA significantly decreased the levels of cyclinD1, CDK4, CDK6 and the phosphorylation of Rb on one hand, and increased the expression of P21 and P27 in a concentration-dependent manner on the other (Fig. 2D and E; *P < 0.05 *versus* control group). These data suggest that TSA inhibits the proliferation of RPE cells by G1 phase cell cycle arrest through inhibition of cyclinD1/CDK4/6 complexes and induction of P21 and P27.

### TSA prevented TGF-β2–induced EMT in RPE cells

**Figure 3:**
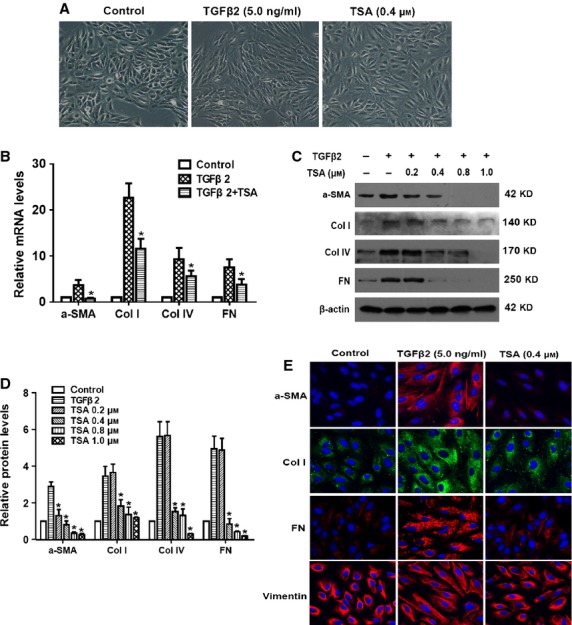
Tricostatin A (TSA) prevents transforming growth factor-β2 (TGF-β2)–induced epithelial–mesenchymal transition in retinal pigment epithelium (RPE) cells. RPE cells were cultured in the absence or presence of TGF-β2 (5 ng/ml) with TSA or DMSO for 24 hrs. (A) Cell morphology was examined using phase contrast microscope under ×100 magnification. (B) The mRNA expression levels of α-SMA, Col I, Col IV and fibronectin (FN) were determined by real-time quantitative PCR. Gene expression levels were normalized to the glyceraldehyde 3-phosphate dehydrogenase control. *P < 0.05 *versus* TGF-β2 treated with DMSO group. (C) The protein expression levels of α-SMA, Col I, Col IV and FN were detected by western blot. (D) Quantification of protein levels from three independent experiments. *P < 0.05 *versus* TGF-β2 treated with DMSO group. (E) Immunofluorescence analysis of α-SMA (red), Col I (green), FN (red) and vimentin (red) using confocal microscopy. Representative images are shown (magnification, ×400).

To explore whether HDAC inhibition could prevent TGF-β2–induced EMT in RPE cells, markers including α-SMA, FN, Col I and IV were investigated at mRNA and protein levels by real-time PCR and western blot respectively. As shown in Figure 3A, TGF-β2 significantly stimulated morphological changes of RPE cells, presenting as marked transition from an epithelial to a greater mesenchymal phenotype. In addition, TGF-β2 significantly increased the expression of α-SMA, Col I, Col IV and FN at both mRNA (Fig. 3B) and protein levels (Fig. 3C and D). In accordance with that, immunofluorescence staining of α-SMA, FN and Col I was also enhanced (Fig. 3E). Vimentin showed no apparent change in fluorescence intensity, but it revealed cytoskeletal reorganization when treated with TGF-β2. Intriguingly, TSA treatment completely abrogated the morphological and cytoskeletal changes of RPE cells, as well as the up-regulation of α-SMA, FN, Col I and Col IV (Fig. 3; *P < 0.05 *versus* TGF-β2 treated with DMSO group). Similarly, we also found that TSA could prevent the TGF-β1–induced morphological changes of RPE cells and the up-regulation of α-SMA, FN and Col IV (Figure S1A–D; *P < 0.05 *versus* TGF-β1 treated with DMSO group). Taken together, these data indicate that HDAC inhibitor TSA can significantly attenuate TGF-β2/TGF-β1–induced EMT in RPE cells.

### TSA abrogated TGF-β2–induced up-regulation of Snail and Slug

**Figure 4:**
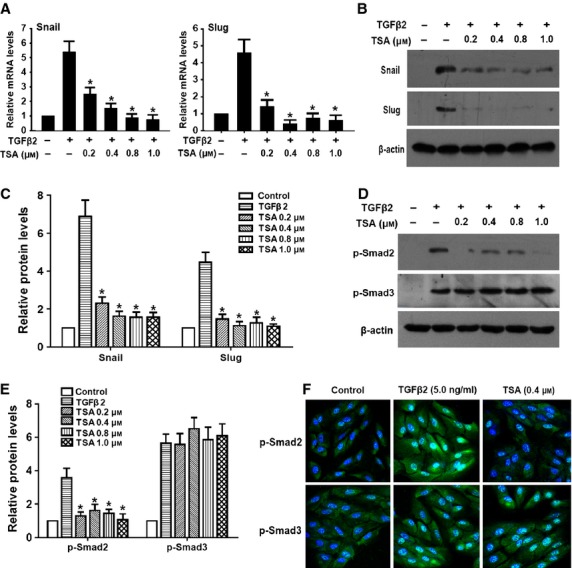
Tricostatin A (TSA) abrogates transforming growth factor-β2 (TGF-β2)–induced up-regulation of Snail and Slug, and phosphorylation of Smad2. Retinal pigment epithelium cells were cultured in the absence or presence of TGF-β2 with TSA (0.2, 0.4, 0.8 and 1.0 μM) or DMSO for 24 hrs, the expression of Snail and Slug was detected by real-time RCR (A) and western blot (B and C) respectively. Gene expression levels were normalized to the glyceraldehyde 3-phosphate dehydrogenase control. *P < 0.05 *versus* TGF-β2 treated with DMSO group. (D) The phosphorylation levels of Smad2 and Smad3 were detected by western blot after 60 min. of treatment. (E) Quantification of protein levels from three independent experiments. *P < 0.05 *versus* TGF-β2 treated with DMSO group. (F) Immunofluorescence analysis of p-Smad2 (green) and p-Smad3 (green) using confocal microscopy. Representative images are shown (magnification, ×400).

Snail and Slug are widely recognized as important regulators in EMT. Activation of Snail and Slug is detected in most known EMT events including development, cancer metastasis and fibrosis 18. Therefore, we next investigated whether the suppressive effects of TSA on RPE cells EMT are mediated *via* regulating the expression of Snail and Slug. As documented in Figure 4A–C, TGF-β2 treatment dramatically induced Snail and Slug at mRNA and protein levels, while TSA treatment abrogated the up-regulation of Snail and Slug in a concentration-dependent manner (*P < 0.05 *versus* TGF-β2 treated with DMSO group). These results suggest that HDACs inhibitors can down-regulate Snail and Slug expression, resulting in the reversal of EMT in RPE cells.

### TSA inhibited TGF-β signalling pathway by suppressing the phosphorylation of Smad2/3

To clarify the mechanism under the inhibitory effect of TSA on TGF-β signal, we investigated the effect of TSA on phosphorylation of Smad2 and Smad3. As shown in Figure 4D–F, TGF-β2 alone induced phosphorylation of Smad2 and Smad3 after treatment for 60 min., whereas co-treatment with TSA inhibited phosphorylation of Smad2, but had no effect on phosphorylation of Smad3. However, we found that TSA dramatically inhibited TGF-β1–induced phosphorylation of both Smad2 and Smad3 simultaneously (Figure S1E and F; *P < 0.05 *versus* TGF-β1 treated with DMSO group). These results suggest that TSA suppresses TGF-β signal through inhibiting phosphorylation of Smad2 and Smad3.

### TSA abrogated TGF-β2–induced EMT through down-regulating the non-canonical TGF-β/Akt, MAPK and ERK1/2 signalling pathways

**Figure 5:**
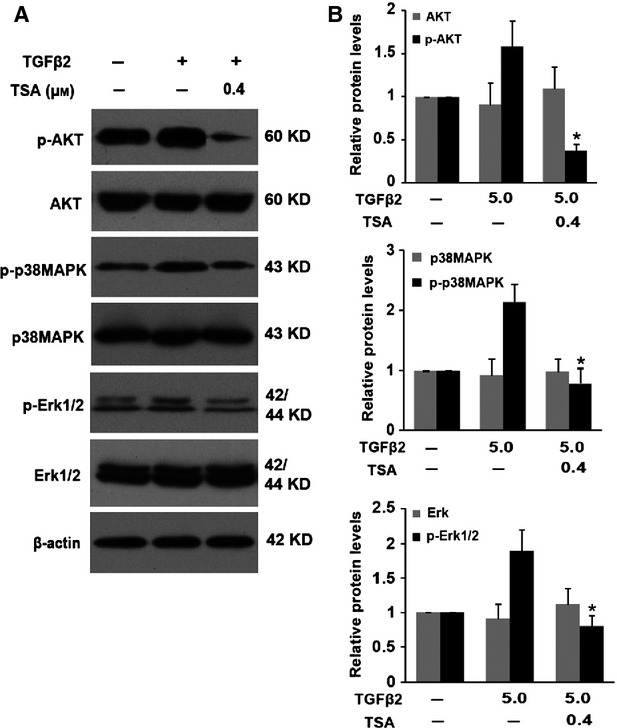
Tricostatin A (TSA) abrogates transforming growth factor-β2 (TGF-β2)–induced epithelial–mesenchymal transition through down-regulating the non-canonical TGF-β/Akt, MAPK and ERK1/2 signaling pathways. (A) Retinal pigment epithelium cells were cultured in the absence or presence of TGF-β2 (5 ng/ml) with TSA (0.4 μM) or DMSO for 60 min., the expression of p-Akt, Akt, p-p38MAPK, p38MAPK, p-ERK1/2 and ERK1/2 was determined by western blot. (B) Quantification of protein levels from three independent experiments. *P < 0.05 *versus* TGF-β2 treated with DMSO group.

The non-canonical Smad signalling, such as the PI3K/Akt, MAPK and ERK1/2, pathways are also activated by TGF-β in different types of cells 19,20; therefore, we also investigated the effect of TSA on TGF-β2–induced activation of Akt, MAPK and ERK1/2 signalling pathways. When RPE cells were stimulated by TGF-β2 for 60 min., Akt, p38MAPK and ERK1/2 were activated *via* phosphorylation, but with the unchanged total protein levels, whereas co-treatment with TSA could attenuate TGF-β2–induced phosphorylation of Akt, p38MAPK and ERK1/2 dramatically (Fig. 5; *P < 0.05 *versus* TGF-β2 treated with DMSO group). These results suggest that TSA abrogates TGF-β2-induced EMT through down-regulating the non-canonical TGF-β/Akt, MAPK and ERK1/2 signalling pathways.

### TSA abrogated TGF-β2–induced EMT via down-regulating the Notch pathway

**Figure 6:**
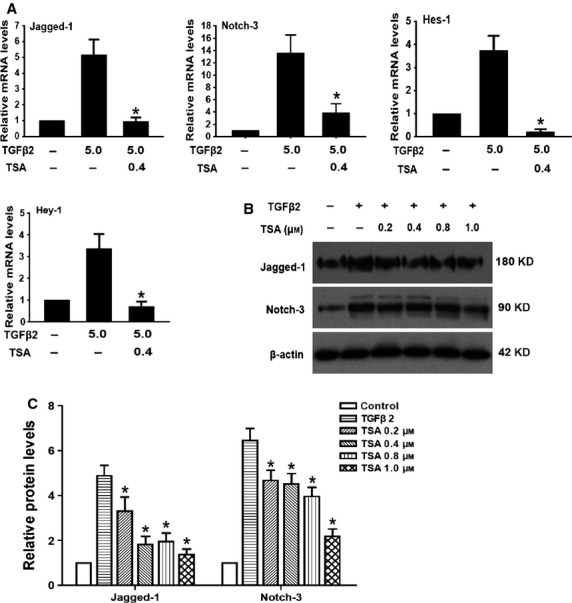
Tricostatin A (TSA) abrogates transforming growth factor-β2 (TGF-β2)–induced epithelial–mesenchymal transition *via* down-regulating the Notch pathway. (A) Retinal pigment epithelium cells were treated with TGF-β2 in the presence of TSA (0.4 μM) or DMSO for 24 hrs; the mRNA expression levels of Jagged-1, Notch-3, Hes-1 and Hey-1 were detected by real-time PCR. Gene levels were normalized to control glyceraldehyde 3-phosphate dehydrogenase. *P < 0.05 *versus* TGF-β2 treated with DMSO group. (B) The protein expression levels of Jagged-1and Notch-3 were detected by western blot. (C) Quantification of protein levels from three independent experiments. *P < 0.05 *versus* TGF-β2 treated with DMSO group.

Emerging evidence suggests that the Notch signalling pathway is a key coordinator in the induction of EMT during embryonic development, fibrotic diseases and cancer metastasis 21. Our previous study also reported that Jagged/Notch pathway is activated during TGF-β2–induced EMT of human RPE cells in association with the induction of Jagged-1 and Notch-3 expression, while blockade of Notch pathway inhibits TGF-β2–induced EMT (X. Chen, W. Xiao, Y. Liu, unpublished data). Therefore, we next investigated whether the effects of TSA on RPE cells EMT are partly mediated *via* regulating the Notch signalling. In accordance with our previous research, TGF-β2 treatment alone significantly increased the expression of Jagged-1 and Notch-3, as well as their downstream genes Hes-1 and Hey-1. In contrast, TSA could completely attenuate the TGF-β2–induced up-regulation of Jagged-1, Notch-3, Hes-1 and Hey-1 (Fig. 6; *P < 0.05 *versus* TGF-β2 treated with DMSO group). These results suggest that TSA abrogates TGF-β2–induced EMT through down-regulating the Jagged/Notch pathway.

## Discussion

Emerging evidence has proven that the development of PVR largely attributes to proliferation and EMT of RPE cells in response to a variety of cytokines, typically TGF-β2. Recently, HDACs, which are found to play important roles in controlling various physiological and pathological events, and HDAC inhibitors exert potently inhibitory effects on several types of human cancer and fibrotic diseases. In this study, we identified for the first time that some of class I and class II HDACs were up-regulated significantly in TGF-β2/TGF-β1–induced EMT in human RPE cells. Inhibition of HDAC activity with TSA strongly inhibited the proliferation and TGF-β2/TGF-β1–induced EMT in RPE cells. Moreover, TSA prevented TGF-β2–induced EMT through regulating not only the canonical Smad signalling pathway but also the non-canonical TGF-β/Akt, MAPK and ERK1/2 pathways. Finally, we also demonstrated that the underlying mechanism of TSA affects EMT also through down-regulating the Jagged/Notch signalling pathway.

Recent reports have indicated that changes in the histone acetylation are correlated with various pathological conditions, and HDAC inhibitors are now considered to be promising anticancer agents, leading to growth arrest, differentiation and apoptosis of cells 12. In this study, we demonstrated that inhibition of HDAC activity with TSA strongly inhibited the proliferation of RPE cells by G1 phase arrest. Next, we wished to determine the underlying molecular mechanism. The progression of cell cycle is governed by complexes containing cyclins and CDKs, which phosphorylate proteins of the retinoblastoma tumour suppressor (Rb) family, and then further promote G1/S phase progression. Besides that, CDKs inhibitors, such as P21 and P27, block cell cycle progression by inhibiting the activity of cyclin/CDKs complexes 22. Consequently, we investigated the expression of cyclinD1, CDK4, CDK6, p-Rb, P21 and P27, which are key to cell cycle regulation. The results from this study provide convincing evidence that TSA exerts its inhibitory effects on cell cycle progression primarily *via* inhibiting cyclinD1/CDK4/6/p-Rb, and inducing P21 and P27.

Emerging evidence is increasingly showing that aberrant expression of HDACs may contribute to the development and progression of various cancers and many other diseases 23,24. In this study, we identified that multiple class I and class II HDACs were up-regulated significantly during TGF-β2/TGF-β1–induced EMT in human RPE cells. Earlier research has reported that HDAC1 is required for TGF-β–induced EMT and cell migration in hepatocytes, and HDAC inhibitors completely suppress TGF-β–induced EMT in murine hepatocytes 15. HDAC2 is also involved in TGF-β–induced EMT in diabetic nephropathy 25. Previous study also revealed that HDAC3 is essential for hypoxia-induced EMT in liver cells 26. However, there is no existing report concerning the role of the other HDACs in EMT. To testify our hypothesis that inhibition of HDACs can abrogate TGF-β–induced EMT in RPE cells, we used TSA, a class I and II HDAC inhibitor. The results showed that inhibition of HDACs is capable of reducing the expression of mesenchymal markers (*i.e*. α-SMA, Col I, Col IV and FN) and preventing cytoskeletal defect. Taken together, these results suggest that HDACs may play critical roles in promoting EMT in RPE cells, and HDAC inhibitor can be useful for abrogating EMT phenotype.

Snail and Slug are believed to play critical roles in the processes of EMT, tumour cell invasion and metastasis. They regulate various aspects of the EMT phenotype, such as decreasing expression of various epithelial markers 27 and increasing expression of mesenchymal cell/fibroblast markers 28. In fact, it has been shown that all known EMT events during development, cancer and fibrosis appear to be associated with Snail activation 18,29. In this study, we demonstrated that TSA significantly down-regulated Snail and Slug expression at both mRNA and protein levels. These results could partly explain the mechanism under the reversal of EMT by inhibiting HDAC activity – inhibition of Snail and Slug expression.

The canonical TGF-β/Smad signalling transmits its signal *via* binding to two related transmembrane type I and type II receptors, which subsequently phosphorylate receptor-regulated Smad proteins – Smad2 and/or Smad3 30. In our study, TSA inhibited TGF-β2–induced phosphorylation of Smad2, but had no effect on Smad3. However, TSA dramatically inhibited TGF-β1–induced phosphorylation of both Smad2 and Smad3 simultaneously. Hence, these results indicate that TSA abrogates TGF-β–induced EMT *via* partly affecting the canonical Smad2/3 signalling.

Previous studies have shown that other non-Smad signallings are also involved in TGF-β–induced EMT in different types of cells, including PI3K/Akt, p38MAPK and ERK1/2 signalling pathways 19,20,31,32. Although TGF-β signalling occupies a central position in the signalling networks that control EMT, recent evidence shows that the MAPK and the PI3K/Akt pathways can cross-talk and integrate with canonical TGF-β/Smad signalling, thereby contributing to EMT 31,33. In RPE cells, we also demonstrated that Akt, p38MAPK and ERK1/2 signalling pathways were activated by TGF-β2, whereas TSA could dramatically attenuate the TGF-β2–induced phosphorylation of Akt, p38MAPK and ERK1/2. In summary, TSA abrogates TGF-β2–induced EMT through not only the canonical TGF-β/Smad pathway but also the non-canonical TGF-β/Akt, MAPK and ERK1/2 signalling pathways.

Recently, the Notch signalling pathway has been proved to be a key regulator in EMT during embryonic development, fibrotic diseases and cancer metastasis 21. Elevated Jagged/Notch signalling has been verified in a large range of fibrotic diseases developed in the kidney, liver and lung 34. Moreover, our previous study also found that the elements of the Notch signalling pathway, including Jagged-1, Notch-3, Hes-1 and Hey-1, were up-regulated in TGF-β2–stimulated EMT in human RPE cells. Furthermore, blockade of Notch pathway inhibited TGF-β2–induced EMT, suggesting a critical role of Notch pathway in TGF-β2–induced EMT (X Chen, W Xiao, X Liu, M Zeng, L Luo, M Wu, S Yeand, Y Liu, unpublished data). In this study, we proved that TSA could attenuate the TGF-β2–induced up-regulation of Jagged-1, Notch-3 and their target genes Hes-1 and Hey-1. Collectively, these results suggest that the inactivation of Jagged/Notch signalling is another potential mechanism under the EMT abrogation by TSA in RPE cells.

In summary, our results provided, for the first time, evidence that the inhibition of HDAC activity with TSA strongly inhibits the proliferation and TGF-β2–induced EMT in human RPE cells. The mechanism underlying EMT suppression by TSA is the inactivation of both canonical Smad and non-Smad pathways, including Akt, p38MAPK and ERK1/2 pathways. Meanwhile, blockade of Jagged/Notch signalling pathway may contribute to the arrest of EMT as well. Therefore, this study provides a new insight into the pathogenesis of PVR in an epigenetic aspect. Epigenetic regulators, represented by HDAC inhibitors, may have therapeutic value in the prevention and treatment of PVR.

## Supporting information

Figure S1 TSA prevents TGF-b1–induced EMT and phosphorylation of Smad2/3 in RPE cells.
