# Supplementary figures and images for: Trichostatin A, a histone deacetylase inhibitor, suppresses proliferation and epithelial–mesenchymal transition in retinal pigment epithelium cells
Source: J Cell Mol Med. 2014 Jan 23;18(4):646–55. doi: 10.1111/jcmm.12212 (PMC4000116; doi:10.1111/jcmm.12212)

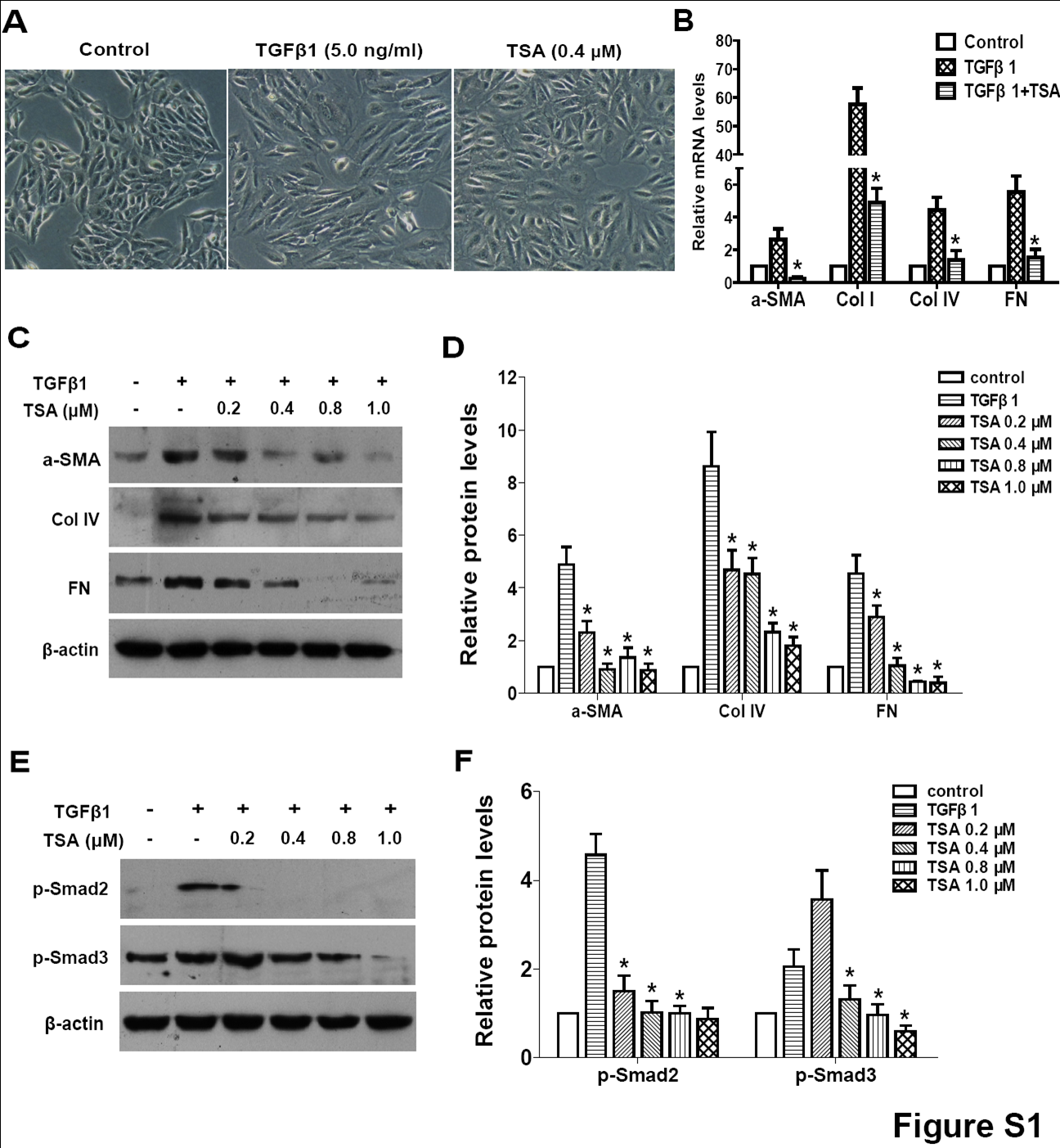

Supplement: Figure S1 [file jcmm0018-0646-sd1.tif]
